# Estimated Incidence and Prevalence of Metastatic Breast Cancer in Northern Ireland, 2009 to 2020

**DOI:** 10.1001/jamanetworkopen.2024.53311

**Published:** 2025-01-06

**Authors:** Sinead Teresa Hawkins, Amisha Ashok, Jackie M. Kelly, Gerard Savage, Deirdre Fitzpatrick, Helen Mitchell, Ann McBrien, Damien Bennett

**Affiliations:** 1 Northern Ireland Cancer Registry, Queen’s University Belfast, Belfast, Northern Ireland, United Kingdom; 2 Centre for Public Health, Queen’s University, Belfast, Northern Ireland, United Kingdom; 3 Metastatic Breast Cancer Patient, Belfast, Northern Ireland, United Kingdom

## Abstract

This cohort study examines the incidence and prevalence of metastatic breast cancer in Northern Ireland using population-based cancer registry data and health records.

## Introduction

The number of people living with metastatic breast cancer (MBC) is unknown; thus, policymakers and health care institutions cannot adequately plan and deliver MBC surveillance and treatment.^1^ Few countries prospectively collect MBC data, while some retrospectively analyze administrative data.^2^ One study^3^ found that MBC may increase over time. Our study links Northern Ireland population-based cancer registry (PBCR) data, Hospital Inpatient Patient Administrative System (PAS) records and General Register Office (GRO) death records to estimate MBC incidence and prevalence in Northern Ireland.

## Methods

This cohort study followed the Strengthening the Reporting of Observational Studies in Epidemiology (STROBE) reporting guideline. Ethical approval was granted by the Office of Research Ethics Committee Northern Ireland and informed consent was not required.

Patients with primary breast cancer (BC) were identified from the Northern Ireland PBCR from 1993 to 2020 and linked to PAS and GRO data for secondary *ICD-O-3* codes C77 to C79. Further MBC cases were identified by free-text mining of GRO cause of death records (eTable 1 in Supplement 1). A rules-based algorithm identified MBC cases as either de novo (stage IV at primary diagnosis), or progressive or recurrent MBC for patients with stage I to III or unknown at primary diagnosis (eFigure 1 in Supplement 1). Additional information can be found in the eMethods in Supplement 1. Sensitivity, specificity, positive predictive values (PPV), and negative predictive values (NPV) were calculated. Estimated MBC incidence and prevalence were reported comparing the inclusion or exclusion of axillary nodes (AN) as an MBC indicator (*ICD-O-3 *C77.3). Patient characteristics included MBC type, sex, age at MBC identification, and deprivation quintile.^4^ MBC data were analyzed from January 2009 to December 2020 using Stata version 16 (StataCorp).

## Results

This study included 3047 MBC cases (3024 females [99.3%]; 1380 aged 60 to 79 years [45.2%]). Algorithm validation using the reference data found high sensitivity (175 of 184 [95.1%]), specificity (837 of 844 [99.2%]), PPV (175 of 182 [96.2%]), and NPV (837 of 846 [98.9%]) when AN were excluded as MBC indicator and decreased specificity (648 of 840 [77.1%]) and PPV (182 of 374 [48.7%]) when AN were included as an MBC indicator. Thus, AN were excluded from final MBC estimates. Between 2009 and 2020 incident MBC cases were stable (mean [SD], 254 [7] per year), while the prevalent MBC population increased from 587 in 2009 to 911 in 2020 (ie, 55% increase) (Figure). In 2020, 607 patients (67%) with prevalent MBC were surviving with progressive or recurrent disease and 304 (33%) with de novo MBC (Table). Most patients were 60 years or older (472 [52%]). Among prevalent MBC cases, 395 patients (43.4%) resided in less deprived areas and 286 (38.0%) resided in more deprived areas (Table). Primary BC incidence increased from a mean (SD) of 1350 (74) cases per year from 2011 to 2015 to 1468 (54) cases from 2016 to 2020; de novo MBC incidence remained stable (mean [SD] of 76 [7] of 1350 cases [5.6%] from 2011 to 2015 vs 79 [9] of 1468 cases [5.4%] from 2016 to 2020).

**Figure.**
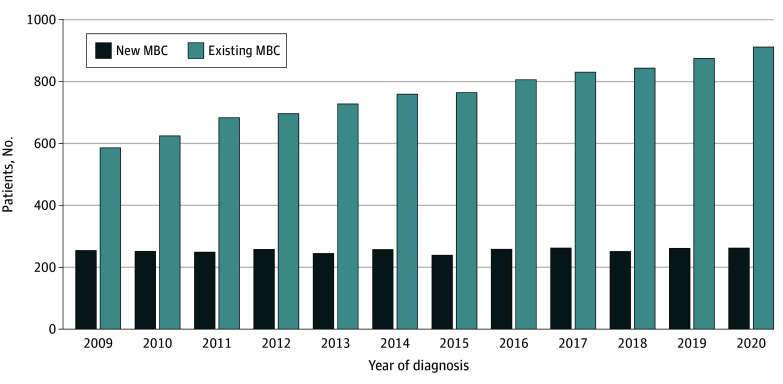
Period-Prevalent Population by New and Existing Cases by Year of Metastatic Breast Cancer (MBC) Diagnosis, 2009-2020

**Table.** Patient Characteristics of Northern Ireland’s Estimated MBC Period Prevalent Population in 2020

Characteristic	Patients, No. (%)
Total MBC population	911 (100.0)
De novo MBC	304 (33.4)
Progressive or recurrent MBC	607 (66.6)
Sex	
Female	905 (99.3)
Male	6 (0.7)
Age at MBC identification, y	
<40	52 (5.7)
40-59	387 (42.5)
60-79	354 (38.9)
≥80	118 (13.0)
Deprivation quintile	
1 (Most deprived)	169 (18.6)
2	117 (19.4)
3	170 (18.7)
4	200 (22.0)
5 (Least deprived)	195 (21.4)

Abbreviation: MBC, metastatic breast cancer.

## Discussion

This novel algorithm accurately identified MBC cases. To our knowledge, this is the first study using PBCR data and validated against a large, reference standard dataset to report estimates of MBC incidence and prevalence for the UK.^2^ We found prevalent MBC cases increased, but excluding AN as an MBC indicator produced lower population estimates.^1,3^ Our estimates of crude incidence and prevalence were similar to prior studies.^5^ There were approximately 250 new MBC cases annually and 911 patients with MBC living in Northern Ireland during 2020. Increasing numbers of patients with MBC has implications for health care planning. Limitations included the exclusion of patients with another primary cancer prior to their primary BC or MBC record; manual review would be required to determine if the metastatic event was associated with the primary BC.

## Notes

Supplement 1.
eMethods.
**eTable. **Example of Terms Used for Free Text Mining of GRO Death Notification Records **eFigure. **Flow Chart of MBC Algorithm Design

Supplement 2.
Data Sharing Statement
