# Supplementary material for: Estimated Incidence and Prevalence of Metastatic Breast Cancer in Northern Ireland, 2009 to 2020
Source: JAMA Netw Open. 2025 Jan 6;8(1):e2453311. doi: 10.1001/jamanetworkopen.2024.53311 (PMC11704970; doi:10.1001/jamanetworkopen.2024.53311)
Supplement: Supplement 1. — eMethods. eTable. Example of Terms Used for Free Text Mining of GRO Death Notification Records eFigure. Flow Chart of MBC Algorithm Design [file jamanetwopen-e2453311-s001.pdf]

Supplemental Online Content

Hawkins ST, Ashok A, Kelly JM, et al. Incidence and prevalence of metastatic breast cancer in Northern Ireland, 2009 to 2020. *JAMA Netw Open*. 2024;8(1):e2453311.
doi:10.1001/jamanetworkopen.2024.53311

eMethods.

eTable. Example of Terms Used for Free Text Mining of GRO Death Notification Records

eFigure. Flow Chart of MBC Algorithm Design

This supplemental material has been provided by the authors to give readers additional information about their work.

© 2024 Hawkins ST et al. *JAMA Netw Open*.

eMethods.

Progressive MBC is the progression of BC to distant metastasis without confirmation of BC remission. Recurrent MBC is the return of BC to a distant site after BC remission. Patients with MBC between 2009 and 2020 were analyzed because MBC codes were stable and stage data were more complete. Results were validated against a reference standard dataset of 1028 patients with BC who were diagnosed in Northern Ireland in 2009 and followed up for MBC via manual health record review until 2017 (184 MBC cases).[1]

1. Cairnduff V, Dwyer L, Lardner S. Investigating characteristics of women with breast cancer recurrence in Northern Ireland (NI). NAACCR/IACR. Accessed November 27, 2024. http://iacr.com.fr/images/AnnualMeetings/Abstracts/2019Vancouver_Book-of-Abstracts.pdf

© 2024 Hawkins ST et al. *JAMA Netw Open*.

eTable 1. Example of Terms Used for Free Text Mining of GRO Death Notification Records

Example of terms used in free text search of GRO 2000-2020 death file.	
metastatic breast cancer<br>metastatic breast carcinoma<br>carcinomatosis<br><br>metastatic carcinoma of breast<br>metastatic breast carcinoma<br>metastatic cancer<br>metastatic lung cancer<br>metastatic liver disease<br>metastatic lung disease<br><br>liver metastases<br>lung metastases<br>brain metastases<br>cerebral metastases<br>bony metastases<br><br>mets<br>bone mets<br>bone metastasis<br>liver mets<br>liver metastasis<br>brain mets<br>brain metastasis<br>lung metastasis<br>lung mets<br><br>secondary carcinomatosis<br>secondary carcinoma<br>secondary breast<br>secondary lung<br>secondary brain<br>secondary liver<br>secondary bone<br>secondary breast<br>secondary breast cancer<br>secondaries<br>breast cancer with secondary spread<br><br>disseminated<br>disseminated carcinomatosis<br>widespread<br><br>breast cancer with metastases to liver and bone<br>breast cancer with metastases to bone and lung<br>carcinoma left breast with metastases in lung and liver	Examples of misspellings<br><br>metastatil breast carcinoma<br>metastic bone disease<br>metastic breast<br>metastic breast cancer<br>metastic carcinoma of breast<br>metastic disease of liver lungs<br>metastic liver disease<br>metaststic breast cancer<br>dessiminated breast carcinoma<br>breast cancer (desseminated)<br>metasatic breast carcinoma<br>metastaic breast carcinoma<br>secandary breast carcinoma<br>metastic disease left breast carcinoma<br>metasatic breast carcinoma<br>cerebral metestases liver and bonne<br>metastic carcinoma<br>breast cancer (desseminated)<br>matastatic breast carcinoma<br>cerebral metastatie cancer

© 2024 Hawkins ST et al. *JAMA Netw Open*.

eFigure. Flow Chart of MBC Algorithm Design

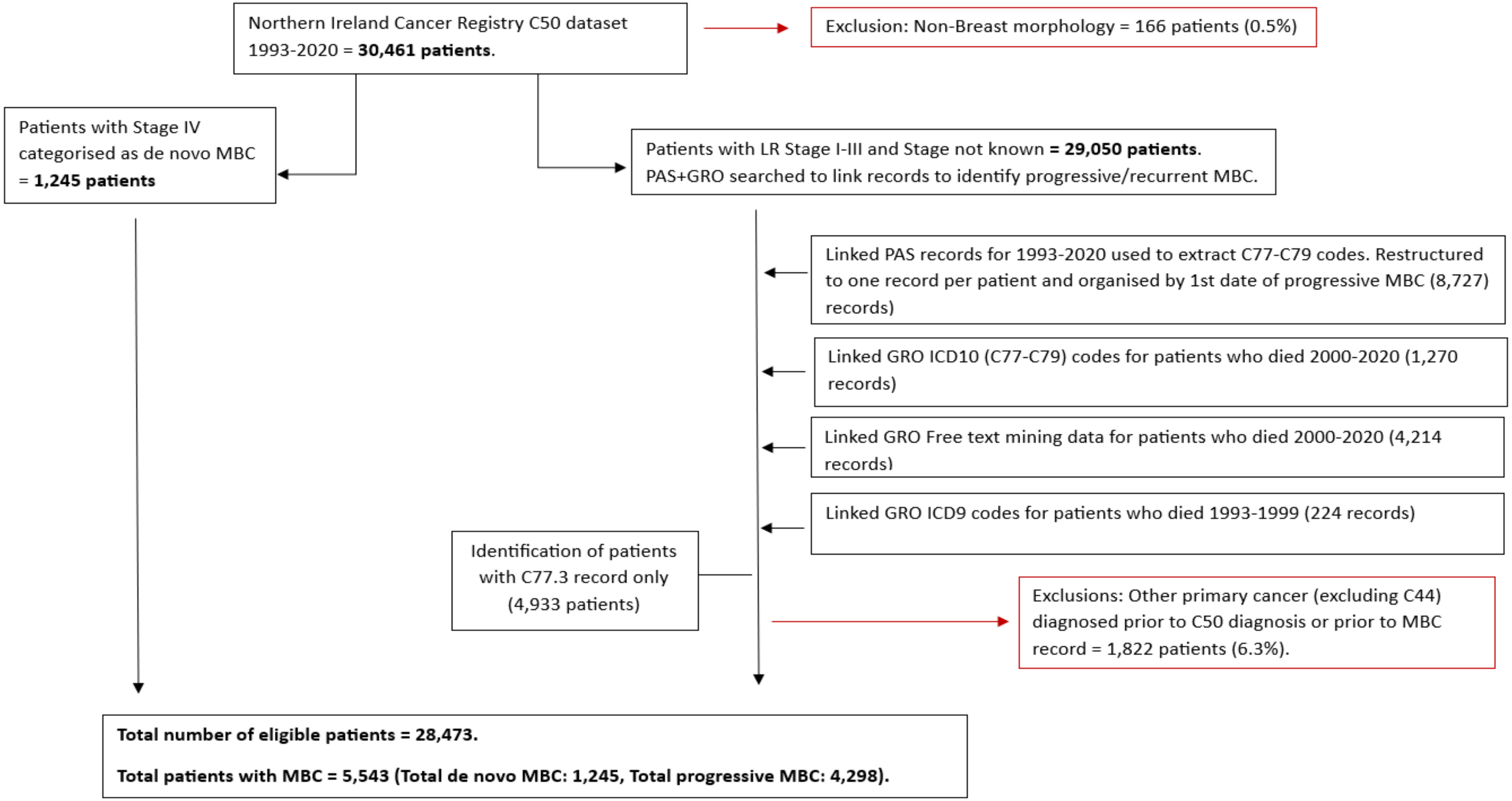

© 2024 Hawkins ST et al. *JAMA Netw Open*.
