# Supplementary material for: Estimated Incidence and Prevalence of Metastatic Breast Cancer in Northern Ireland, 2009 to 2020
Source: JAMA Netw Open. 2025 Jan 6;8(1):e2453311. doi: 10.1001/jamanetworkopen.2024.53311 (PMC11704970; doi:10.1001/jamanetworkopen.2024.53311)
Supplement: Supplement 2. — Data Sharing Statement [file jamanetwopen-e2453311-s002.pdf]

Data Sharing Statement

Hawkins. Estimated Incidence and Prevalence of Metastatic Breast Cancer in Northern Ireland, 2009 to 2020. *JAMA Netw Open*. Published January 06, 2025.
doi:10.1001/jamanetworkopen.2024.53311

Data
Data available: No

Additional Information
Explanation for why data not available: The datasets used in the study are not available publicly due to the need to maintain patient confidentiality. However, study data can be accessed via a secure facility
